# The millipede family Paradoxosomatidae in the Philippines, with a description of *Eustrongylosoma penevi* sp.n., and notes on *Anoplodesmus anthracinus* Pocock, 1895, recorded in Malaysia and Sri Lanka for the first time (Diplopoda, Polydesmida)

**DOI:** 10.3897/BDJ.1.e957

**Published:** 2013-09-16

**Authors:** Sergei Golovatch, Pavel Stoev

**Affiliations:** † Institute for Problems of Ecology and Evolution, Russian Academy of Sciences, Moscow, Russia; ‡ National Museum of Natural History, Bulgarian Academy of Sciences, Sofia, Bulgaria; § Pensoft Publishers, Sofia, Bulgaria

**Keywords:** Millipedes, checklist, Luzon, State Pulau Penang, new species, new record

## Abstract

The Philippine fauna of the family Paradoxosomatidae is reviewed and shown to comprise only 12 certain species (+ one dubious), definitely only a fraction of the real diversity to be expected from such a large tropical archipelago. Two new combinations are proposed: *Euphyodesmus philippina* (Nguyen Duc & Sierwald, 2010), comb. n. ex *Desmoxytes* Chamberlin, 1923, and *Luzonomorpha polilloensis* (San Juan & Lit, 2010), comb. n. ex *Prionopeltis* Pocock, 1895. The first representative of the large, basically Papuan genus *Eustrongylosoma* Silvestri, 1896 is described from Luzon, Philippines: *Eustrongylosoma penevi* sp. n. It differs from the other congeners in certain details of gonopod structure, as well as by the particularly long legs. Based on a restudy of the types of *Strongylosoma luzoniense* Peters, 1864, from Luzon, the species is shown to be a new senior subjective synonym of *Helicorthomorpha orthogona* (Silvestri, 1898), syn. n. This formally results also in *Helicorthomorpha luzoniensis* (Peters, 1864), comb. n. *Anoplodesmus anthracinus* Pocock, 1895 is illustrated and briefly redescribed, based on material from State Pulau Penang, Malaysia, which represents the first formal record of the species in that country. This species is also new to the fauna of Sri Lanka. A review of the *Anoplodesmus* species reported from Sri Lanka, nearly all of them dubious, is presented.

## Introduction

The family Paradoxosomatidae is one of the largest and the most diverse in Diplopoda, and it has long been known to dominate the fauna of the Indo-Australian region (Jeekel 1968). However, only very few paradoxosomatid species have hitherto been recorded in the Philippines. Among them is an unidentified species of the large, mostly Papuan genus *Eustrongylosoma* Silvestri, 1896, reported by Hoffman (1978) from Mindanao. The present paper reviews and updates the paradoxosomatid fauna of the Philippines, including a description of the first Philippine *Eustrongylosoma*. The checklist presented below is highly condensed and skeletal on purpose, because a complete catalogue of the Diplopoda of the Philippines is in preparation (Korsós, pers. comm.). Here we also provide descriptive notes on the Southeast Asian species *Anoplodesmus anthracinus* Pocock, 1895, hitherto known only from Myanmar, but currently found in Malaysia and Sri Lanka as well.

Most of the material is housed in the collection of Diplopoda of the National Museum of Natural History, Sofia (NMNHS), with only a single paratype of *Eustrongylosoma penevi* sp. n. donated to the Zoological Museum, Moscow State University, Russia (ZMUM), as indicated hereafter.

## Taxon treatments

### Eustrongylosoma penevi

Golovatch & Stoev, 2013 sp. n.

urn:lsid:zoobank.org:act:3A600B5D-4791-44CE-AC7C-89BF768789D9

#### Materials

- **Type status:** Holotype. **Occurrence:** recordedBy: P. Stoev & L. Penev; individualCount: 1; sex: male; **Location:** island: Luzon Island; country: Philippines; stateProvince: Mountain Province; verbatimLocality: Mt Polis Checkpoint on the road Banaue – Sagada; verbatimElevation: 1800-1900 m; locationRemarks: under stones and logs; verbatimLatitude: 16°57'58"N; verbatimLongitude: 121°1'37"E; **Event:** eventDate: 6 July 2012; **Record Level:** institutionCode: NMNHS
- **Type status:** Paratype. **Occurrence:** recordedBy: P. Stoev & L. Penev; individualCount: 4; sex: male; **Location:** island: Luzon Island; country: Philippines; stateProvince: Mountain Province; verbatimLocality: Mt Polis Checkpoint on the road Banaue – Sagada; verbatimElevation: 1800-1900 m; locationRemarks: under stones and logs; verbatimLatitude: 16°57'58"N; verbatimLongitude: 121°1'37"E; **Event:** eventDate: 6 July 2012; **Record Level:** institutionCode: NMNHS
- **Type status:** Paratype. **Occurrence:** recordedBy: P. Stoev & L. Penev; individualCount: 1; sex: female; **Location:** island: Luzon Island; country: Philippines; stateProvince: Mountain Province; verbatimLocality: Mt Polis Checkpoint on the road Banaue – Sagada; verbatimElevation: 1800-1900 m; locationRemarks: under stones and logs; verbatimLatitude: 16°57'58"N; verbatimLongitude: 121°1'37"E; **Event:** eventDate: 6 July 2012; **Record Level:** institutionCode: NMNHS
- **Type status:** Paratype. **Occurrence:** recordedBy: P. Stoev & L. Penev; individualCount: 1; lifeStage: juvenile; **Location:** island: Luzon Island; country: Philippines; stateProvince: Mountain Province; verbatimLocality: Mt Polis Checkpoint on the road Banaue – Sagada; verbatimElevation: 1800-1900 m; locationRemarks: under stones and logs; verbatimLatitude: 16°57'58"N; verbatimLongitude: 121°1'37"E; **Event:** eventDate: 6 July 2012; **Record Level:** institutionCode: NMNHS
- **Type status:** Paratype. **Occurrence:** recordedBy: P. Stoev & L. Penev; individualCount: 1; sex: male; **Location:** island: Luzon Island; country: Philippines; stateProvince: Mountain Province; verbatimLocality: Mt Polis Checkpoint on the road Banaue – Sagada; verbatimElevation: 1800-1900 m; locationRemarks: under stones and logs; verbatimLatitude: 16°57'58"N; verbatimLongitude: 121°1'37"E; **Event:** eventDate: 6 July 2012; **Record Level:** institutionCode: ZMUM

#### Description

Length 18-22 (♂) or 23 mm (♀), width of midbody pro- and metazona 1.1-1.3 and 1.5-1.7 mm (♂), or 1.9 and 2.1 mm (♀), respectively. Holotype *ca* 22 mm long, width of pro- and metazona 1.3 and 1.6 mm, respectively. Coloration black to light grey-brown (Fig. 1a). Pattern mostly cingulate due to a large light grey band on prozona dorsally in front of stricture extending down until level of paraterga (Fig. 1a, b). Legs light grey-brown. Antennae increasingly infuscate distad, from light brown to blackish (Fig. 1a).

Body submoniliform. Antennomeres 2 to 6 subequal in length, antennae rather short, reaching behind segment 3 (♂) or 2 (♀) when stretched dorsally. Tegument generally smooth and shining. In width, segments 2 and 3 < collum = 4 < 5-17, thereafter body gently tapering towards telson. Paraterga (Fig. 1b, c, d) modestly developed, keel-shaped, set low (at about 1/3^rd^ of metazonal height), thinner in poreless, thicker in pore-bearing, segments, slightly reaching behind tergal margin only in segments 2 and 17-19, mostly slightly pointed, delimited by a complete and deep sulcus only dorsally, ventral sulcus being incomplete, developed only in posterior quarter to 1/5^th^ extent. Head densely setose on clypeus and frons, bare on vertex. Collum semilunar, bearing two transverse rows of 2+2 setae, one row along front margin, second row in the middle; lateral edges broadly rounded. Metaterga faintly rugulose, a little more clearly so in postsulcus halves, surface below paraterga microgranular in segments 2-7. Tergal setae rather long, about 1/5^th^ of metatergal length, arranged in two rows of 2+2 in each, one in front of, second behind sulcus. Sulcus starting from segment 5, deep, almost reaching the bases of paraterga. Stricture between pro- and metazona finely and densely ribbed. Ozopores lying close to caudal end of paraterga in a shallow ovoid groove, lateral, only partly visible from above. Pleurosternal carinae poorly developed ridges visible only in segments 2-4. Seta at about midway of each paratergum mostly broken off. Axial line wanting. Epiproct (Fig. 2a) subtruncate, pre-apical lateral papillae small. Hypoproct (Fig. 2b) semi-circular. Sternal lobe between coxae 4 subtrapeziform, densely setose (Fig. 2c). Legs very long and slender, about 2.0 (♂) or 1.3 (♀) times as long as midbody height; ♂ tarsal brushes traceable until about legs of segment 15, thereafter thinning out.

Gonopods rather simple (Fig. 2d, e, f): coxite long, subcylindrical, bare; prefemoral portion small, about 1/3^rd^ as long as femorite, the latter slender, ventral lobe somewhat better developed than dorsal one, apicolateral lobe (**l**) rounded, well developed, with a long transverse spine (**s**) at base. Solenophore subcircular, with a subterminal lobule.

#### Diagnosis

Most similar to *Eustrongylosoma exiguum* Hoffman, 1978, from Papua New Guinea, and *Eustrongylosoma kuekenthali* (Attems, 1897), from Borneo and Sulawesi, sharing the presence of a prominent distal spine on the gonopod femorite. Different from all congeners by the transverse orientation of the spine and noticeably long legs in the male (Hoffman 1978, Golovatch 1997).

#### Etymology

Honours our good friend and colleague Lyubomir Penev, biologist and founder of the Biodiversity Data Journal and Pensoft Publishers.

#### Notes

**Figure 3.**
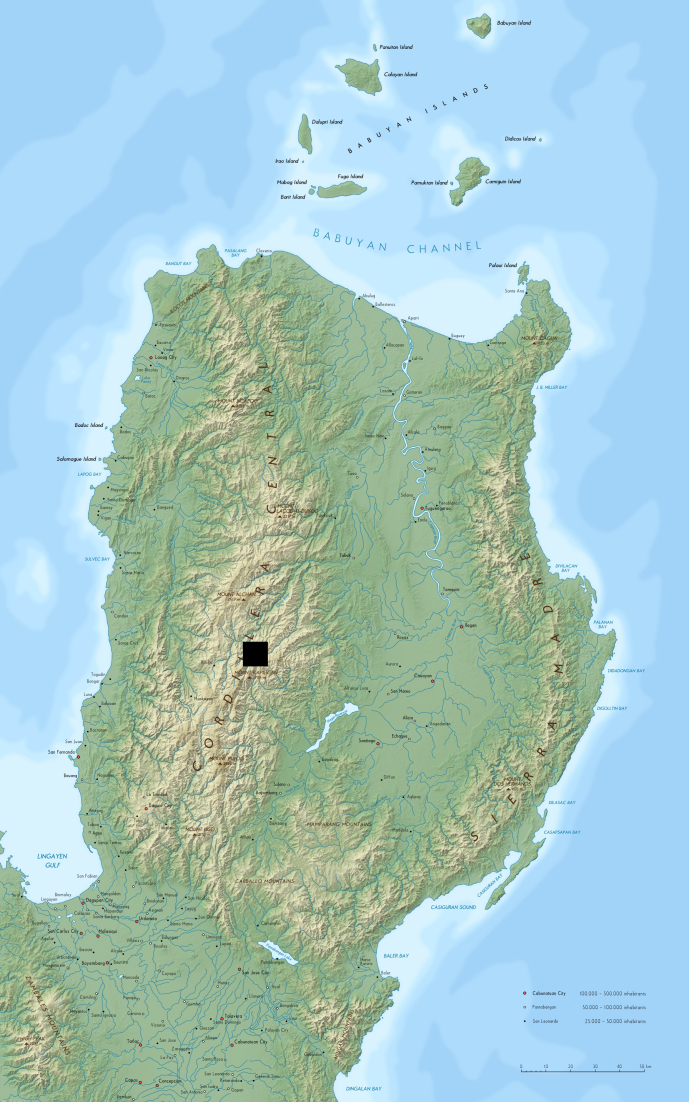
Map of Luzon showing the type locality of *Eustrongylosoma penevi* sp. n. Original map from: Alexander Altenhof (Kater Begemot) via Wikimedia Commons.

**Figure 4.**
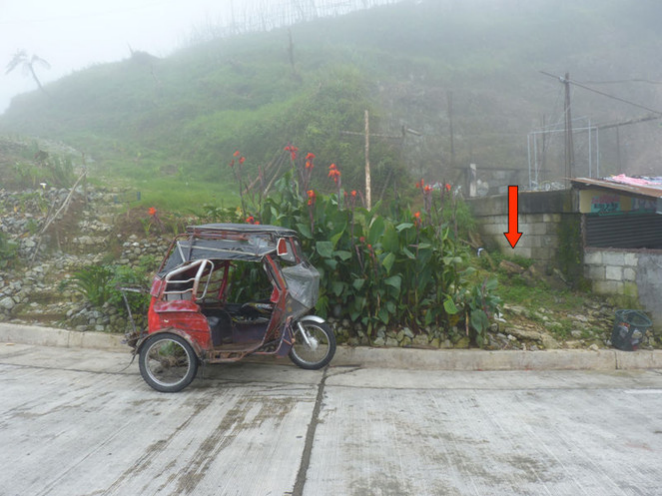
A view of Mt Polis Checkpoint, the type locality of *Eustrongylosoma penevi* sp. n. Red arrow indicates the exact place where species was found.

The species is hitherto known only from its type locality, Mt Polis Checkpoint on the road Banaue – Sagada (Fig. 3), where it was found close to a human settlement, under wooden plates and logs (Fig. 4).

### Anoplodesmus anthracinus

Pocock, 1895

#### Materials

- **Type status:** Other material. **Occurrence:** recordedBy: P. Stoev & L. Penev; individualCount: 1; sex: male; behavior: copulation observed; **Location:** higherGeography: Malay Peninsula; country: Malaysia; stateProvince: State Pulau Penang; verbatimLocality: Station MARDI Seberang Perai; locationRemarks: agriculture area, in close proximity to experimental rice fields, under wooden board; verbatimLatitude: 5°32'24''N; verbatimLongitude: 100°28'11''E; **Event:** eventDate: 15 June 2011; **Record Level:** institutionCode: NMNHS
- **Type status:** Other material. **Occurrence:** recordedBy: P. Stoev & L. Penev; individualCount: 2; sex: females; behavior: copulation observed; **Location:** higherGeography: Malay Peninsula; country: Malaysia; stateProvince: State Pulau Penang; verbatimLocality: Station MARDI Seberang Perai; locationRemarks: agriculture area, in close proximity to experimental rice fields, under wooden board; verbatimLatitude: 5°32'24''N; verbatimLongitude: 100°28'11''E; **Event:** eventDate: 15 June 2011; **Record Level:** institutionCode: NMNHS
- **Type status:** Other material. **Occurrence:** recordedBy: I. Melnik; individualCount: 1; sex: male; **Location:** country: Sri Lanka; stateProvince: Sabaragamuwa Prov.; verbatimLocality: Millenium Foundation Orphanage; verbatimElevation: 90 m; verbatimLatitude: 7°16'40"N; verbatimLongitude: 80°23'12"E; **Event:** eventDate: 19-22.XII.2012; **Record Level:** institutionCode: ZMUM
- **Type status:** Other material. **Occurrence:** recordedBy: I. Melnik; individualCount: 2; sex: females; **Location:** country: Sri Lanka; stateProvince: Sabaragamuwa Prov.; verbatimLocality: Millenium Foundation Orphanage; verbatimElevation: 90 m; verbatimLatitude: 7°16'40"N; verbatimLongitude: 80°23'12"E; **Event:** eventDate: 19-22.XII.2012; **Record Level:** institutionCode: ZMUM

#### Description

Length *ca* 33 mm, width of pro- and metazona 2.8 and 3.8 mm, respectively (♂), or 25, 3.0 and 4.0 mm, respectively (♀). Colour pattern highly vivid (Fig. 5), shiny blackish to dark brown, with contrasting yellowish paraterga and the immediately adjacent regions. Paraterga very well-developed, set rather high (about 1/4^th^ of midbody height) (Fig. 6a), callus wide (Fig. 6b), thicker in pore-bearing paraterga. Pleurosternal carinae longitudinally arched ribs, increasingly poorly developed towards telson to totally disappear in segment 15. Legs only slightly enlarged in male, rather long and slender, about 1.3 (♂) or 0.9 (♀) times as long as midbody height. ♂ legs 5 (Fig. 7a) and 6 with large femoral humps, ♂ femur 7 with a humped process even greater than in leg 6 (Fig. 7b). Epiproct subtruncate (Fig. 6c). Hypoproct roundly subtrapeziform (Fig. 6d). Sternal lamina between ♂ coxae 4 semi-circular (Fig. 6e).

Gonopods very simple (Fig. 6f): coxite with a few strong setae distodorsally, prefemoral part prominent, only slightly shorter than acropodite; femorite with a strong ventral tooth (**a**), solenophore bipartite, with two apical lobes (**b**, **c**), lobe **c** supporting a short solenomere (**sl**).

#### Notes

This species was originally described from Yangon (= Rangoon), Myanmar (Pocock 1895). Attems (1937) synonymized it with *Jonespeltis splendidus* Verhoeff, 1936, from southern India, but Jeekel (1965) revalidated the latter species and returned *Anoplodesmus anthracinus* to its original scope. Furthermore, Jeekel provided very useful illustrations and a detailed redescription of the species, based on a part of the type series. Hoffman (1973) gave more illustrations of the gonopods, based on a paratype of *Anoplodesmus kathanus* (Chamberlin, 1921), from Katha, north of Yangon, Myanmar, and synonymized it with *Anoplodesmus anthracinus*.

Our record of *Anoplodesmus anthracinus* in the State of Pulau Penang, Malaysia considerably extends the range of this species to the south. The studied sample agrees well with the description provided by Jeekel (1965) and Hoffman (1973) in most characters (Figs 6f, 7), including humps in ♂ femora 5 and 6, as well as a process surmounting a hump in ♂ femur 7. Only slight variations have been noticed in the shapes of paraterga and sternal lobe between ♂ coxae 4. The same can be said about the samples from Sri Lanka which are also identified as *Anoplodesmus anthracinus*.

**Figure 8.**
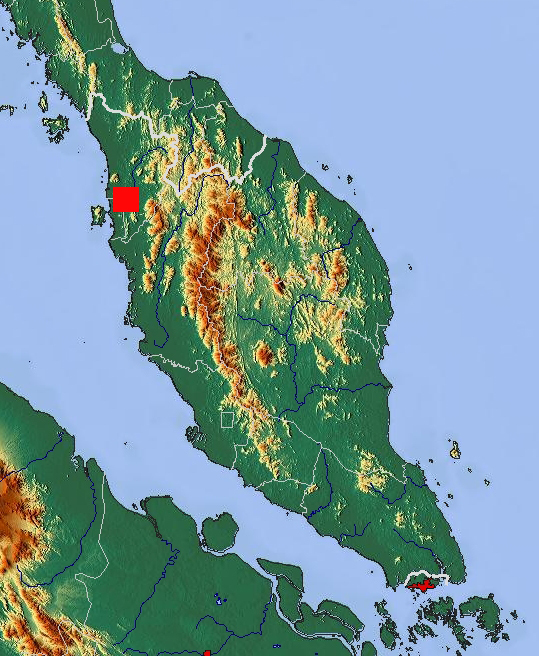
Map of the southern part of Malay Peninsula showing the new locality of *Anoplodesmus anthracinus*. Original map from Wikimedia Commons.

**Figure 9.**
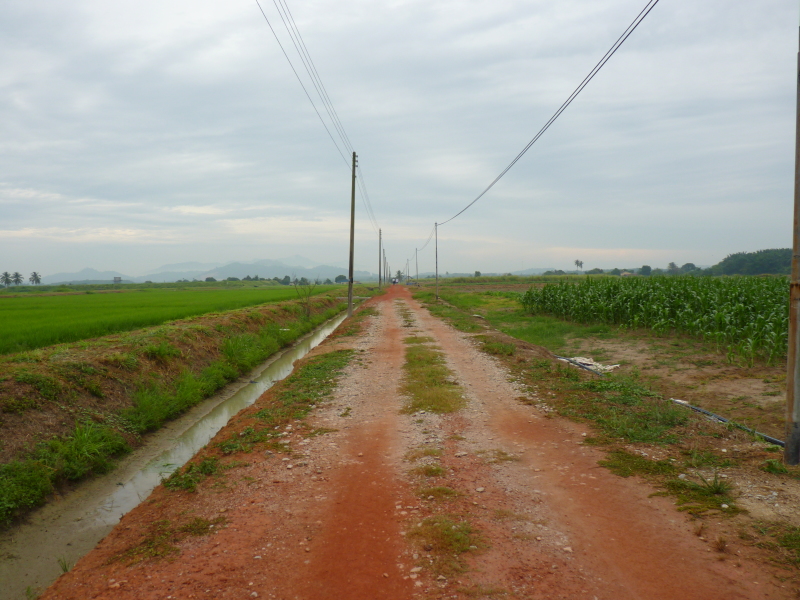
A view of Station MARDI Seberang Perai where *Anoplodesmus anthracinus* was found.

**Figure 10.**
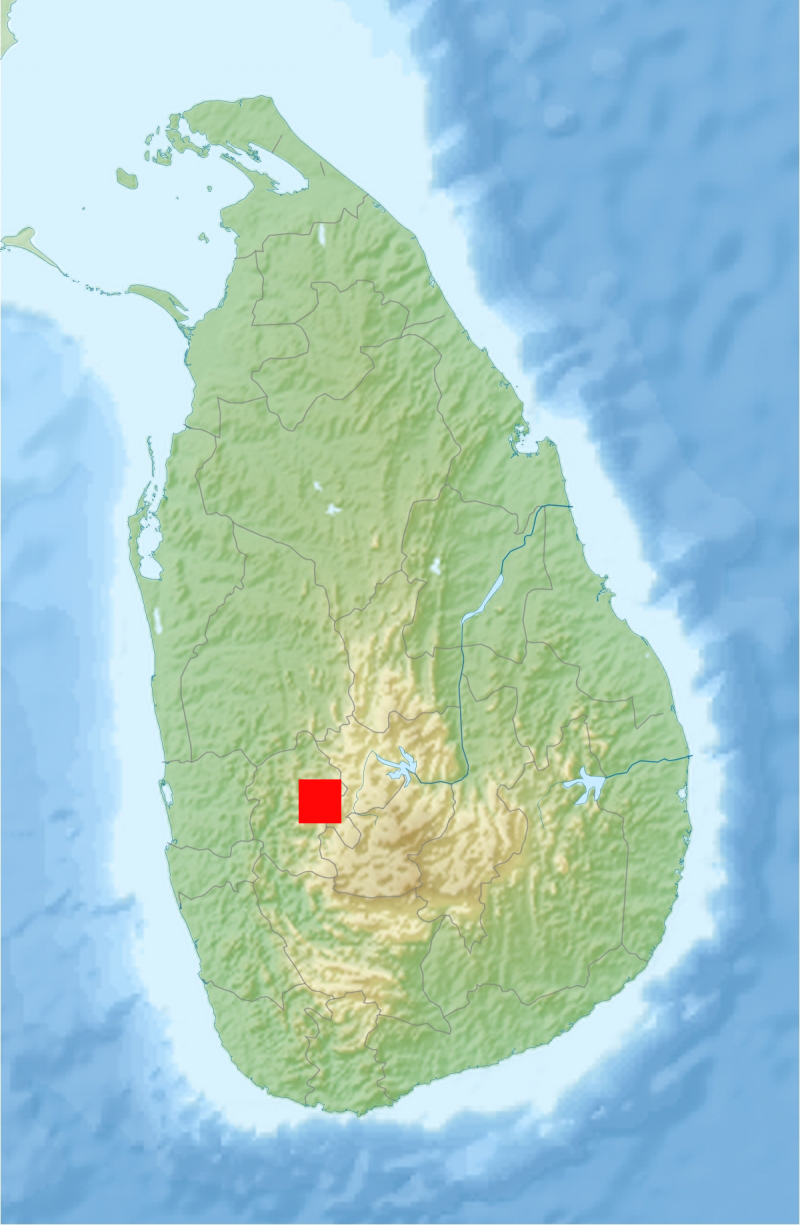
Map of Sri Lanka showing the new locality of *Anoplodesmus anthracinus*. Original map from: Uwe Dedering, via Wikimedia Commons.

These are the first formal records of the species in Malaysia and Sri Lanka (Figs 8, 10). However, actually they might well represent introductions. In fact, in Malaysia the species was observed and collected in a highly agricultural and urbanized area, in close proximity to experimental rice fields (Fig. 9), while in Sri Lanka, the collecting locality is a human settlement.

It is noteworthy that Sri Lanka hosts several formal species of *Anoplodesmus*, nearly all very similar to one another:

- *Anoplodesmus saussurii* (Humbert, 1865), originally described from Sri Lanka, later recorded also in Fiji and Mauritius (Jeekel 1965, Jeekel 1972, Jeekel 1980a). The only meaningful difference from *Anoplodesmus anthracinus* is said to lie in the absence of a ventral hump in ♂ femur 5. However, given considerable variation in the presence or absence of this character, when such a hump in *Anoplodesmus anthracinus* can either be present in or absent from ♂ femur 4 (Attems 1937, Jeekel 1965), its status versus the older name *Anoplodesmus saussurii* is to be questioned.
- *Anoplodesmus luctuosus* (Peters, 1864), from Rambodde; *Anoplodesmus inornatus* (Humbert, 1865), *Anoplodesmus layardi* (Humbert, 1865), *Anoplodesmus thwaitesii* (Humbert, 1865) and *Anoplodesmus humberti* (Carl, 1902), all from Paradeniya; and *Anoplodesmus sabulosus* Attems, 1898, from Kandy. All of them have been described from Sri Lanka, still known only from that island. Some of these taxa are however dubious, being based on female or even juvenile material, but most could be included into a key (Jeekel 1965). Regrettably, the first couplet in the key is purely geographic, separating the species from Myanmar and Sumatra from those described from Sri Lanka and India (Jeekel 1965). As one can see from the presently known distributions of *Anoplodesmus saussurii* and *Anoplodesmus anthracinus*, this distinction does not hold, also strongly suggesting several introductions through human agency. The only feasible solution lies in collecting new and/or spotting topotypic museum samples of the still enigmatic *Anoplodesmus inornatus* and *Anoplodesmus layardi* from Paradeniya, and of *Anoplodesmus sabulosus* from Kandy, to properly compare them to their type material. In addition, bar-coding could help tracing genetic relationships. Last, but not least, a few congeneric species, most of which also very similar to *Anoplodesmus anthracinus*, are known to occur in southern India as well.
- Since *Anoplodesmus* is a senior synonym of *Paranedyopus* (Golovatch 2000, Golovatch 2013), the sole erstwhile component species of the latter genus from Sri Lanka, *Anoplodesmus simplex* (Humbert, 1865), from Pundaloya (Jeekel 1980c), must be considered as well. However, like any former *Paranedyopus* species, *Anoplodesmus simplex* shows reduced paraterga and more elaborate gonopods (Golovatch 2013). In other words, *Anoplodesmus simplex* is quite distinct from the above congeners from Sri Lanka which all have strongly developed paraterga and highly simple gonopods. In contrast, it seems to be more similar to *Anoplodesmus rufocinctus* (Carl, 1932) and *Anoplodesmus subcylindricus* (Carl, 1932), both latter taxa from southern India (Jeekel 1980c).
- *Anoplodesmus anthracinus*, new to the fauna of Sri Lanka.

## Checklists

### Checklist of the Philippine Paradoxosomatidae

#### Australiosomatinae

#### Antichiropodini

#### Euphyodesmus

Attems, 1931

Euphyodesmus Type-species: *Euphyodesmus gracilis* Attems, 1931

#### Euphyodesmus philippina

(Nguyen Duc & Sierwald, 2010), comb. n.

##### Notes

The identity of this species, described from Palawan Island in the genus *Desmoxytes* Chamberlin, 1923 (Nguyen Duc and Sierwald 2010), has recently been discussed and shown to actually represent the basically Australian subfamily Australiosomatinae (Golovatch et al. 2012). The species has thereby remained referred to as *“Desmoxytes” philippina*, the genus name being put in quotation marks to emphasize the wrong original assignment. Here we take the opportunity to allocate it properly at least at the subfamily level, choosing the Bornean *Euphyodesmus* as perhaps the best candidate genus (Golovatch 1996).

#### Paradoxosomatinae

#### Orthomorphini

#### Luzonomorpha

Hoffman, 1973

Luzonomorpha Type-species: *Prionopeltis montana* Chamberlin, 1921

##### Notes

This strictly Philippine genus has recently been reviewed, and most of its species have been keyed (Jeekel 2000).

#### Luzonomorpha acutangula

(Newport, 1844)

##### Notes

Described as *Polydesmus acutangulus* from an unspecified locality in the Philippines, it has sometimes been quoted in the original spelling (e.g. Jeekel 2000), but we prefer to follow Hoffman (1973), who clearly spelled the name in the feminine gender.

#### Luzonomorpha infulata

(Wang, 1951)

##### Notes

Described from Mindanao Island.

#### Luzonomorpha montana

(Chamberlin, 1921)

##### Notes

Described from several places in Luzon.

#### Luzonomorpha pallidula

Jeekel, 2000

##### Notes

Known from Mindoro Island.

#### Luzonomorpha picea

(Brandt, 1839)

##### Notes

Described from Manila.

#### Luzonomorpha polilloensis

(San Juan & Lit, 2010), comb. n.

##### Notes

Known from Polillo Island (San Juan and Lit 2010). This species was originally assigned to *Prionopeltis* Pocock, 1895, a genus long known to be invalid (Jeekel 1968). In fact it definitely belongs to *Luzonomorpha*.

#### Luzonomorpha quatuorputeus

(Wang, 1951)

##### Notes

Known both from Mindanao and Luzon islands.

#### Orthomorpha

Bollman, 1893

Orthomorpha Type-species: *Polydesmus beaumontii* Leguillou, 1841

#### Orthomorpha coarctata

(De Saussure, 1860)

##### Notes

This pantropical, definitely anthropochore species has often been referred to as a distinct genus, *Asiomorpha* Verhoeff, 1939, but we prefer to regard *Orthomorpha coarctata* as a species of *Orthomorpha* (see Likhitrakarn et al. 2011). In the Philippines, it has been recorded in Mactan and Cebu islands (Wang 1955).

#### Sulciferini

#### Chondromorpha

Silvestri, 1897

Chondromorpha Type-species: *Chondromorpha severini* Silvestri, 1897

#### Chondromorpha xanthotricha

(Attems, 1898)

##### Notes

In the Philippines, this nearly pantropical, definitely anthropochore species has only been recorded in Luzon (Wang 1953).

#### Eustrongylosomatini

#### Eustrongylosoma

Silvestri, 1896

#### Eustrongylosoma penevi

Golovatch & Stoev, 2013, sp. n.

##### Notes

Known from Luzon Island.

#### Tectoporini

#### Helicorthomorpha

Attems, 1914

Helicorthomorpha Type-species: *Strongylosoma holstii* Pocock, 1895

#### Helicorthomorpha luzoniensis

(Peters, 1864), comb. n.

##### Notes

Originally described from Luzon, without a more precise locality (Peters 1864), this species, previously considered dubious (Jeekel 1968), appears to actually represent a new senior subjective synonym of *Helicorthomorpha orthogona* (Silvestri, 1898). The syntypes (1 ♂, 1 ♀), labelled "Bosoboso, Luzon, leg. Martens" (Moritz and Fischer 1978), have been revised and returned to the Museum für Naturkunde in Berlin as а lectotype (♂) and а paralectotype (♀). Lectotype designation is necessary to ensure the species to be based on male material. In addition, unlike the paralectotype, which is an incomplete female, the lectotype is complete. The name *luzoniensis* is preferred because of its priority, being in use in the last 50 years and thus not representing a *nomen oblitum* (Moritz and Fischer 1978). This widespread species, previously referred to as *Helicorthomorpha orthogona*, is known to occur from China to New Guinea (Jeekel 2009). In the Philippines it has been recorded from Luzon and Mindanao islands (Wang 1951, Jeekel 1980b).

### Species incertae sedis

#### Orthomorpha bisulcata

Pocock, 1895

##### Notes

This species, originally described from Myanmar (Pocock 1895), has been recorded under this name from the Philippines, based on material shipped from the Philippines and intercepted by quarantine in the Hawaiis (Wang 1951). Because that material contained only females, the identification seems to be highly dubious, better to be ignored altogether.

## Supplementary Material

XML Treatment for Eustrongylosoma penevi

XML Treatment for Anoplodesmus anthracinus

XML Treatment for Australiosomatinae

XML Treatment for Antichiropodini

XML Treatment for Euphyodesmus

XML Treatment for Euphyodesmus philippina

XML Treatment for Paradoxosomatinae

XML Treatment for Orthomorphini

XML Treatment for Luzonomorpha

XML Treatment for Luzonomorpha acutangula

XML Treatment for Luzonomorpha infulata

XML Treatment for Luzonomorpha montana

XML Treatment for Luzonomorpha pallidula

XML Treatment for Luzonomorpha picea

XML Treatment for Luzonomorpha polilloensis

XML Treatment for Luzonomorpha quatuorputeus

XML Treatment for Orthomorpha

XML Treatment for Orthomorpha coarctata

XML Treatment for Sulciferini

XML Treatment for Chondromorpha

XML Treatment for Chondromorpha xanthotricha

XML Treatment for Eustrongylosomatini

XML Treatment for Eustrongylosoma

XML Treatment for Eustrongylosoma penevi

XML Treatment for Tectoporini

XML Treatment for Helicorthomorpha

XML Treatment for Helicorthomorpha luzoniensis

XML Treatment for Orthomorpha bisulcata

## Figures and Tables

**Figure 1a.**
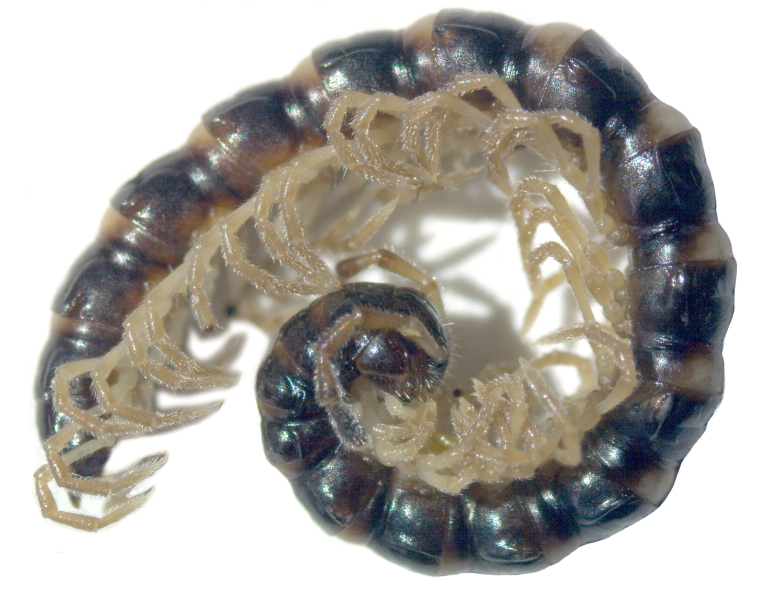
♂ paratype, habitus, lateral view

**Figure 1b.**
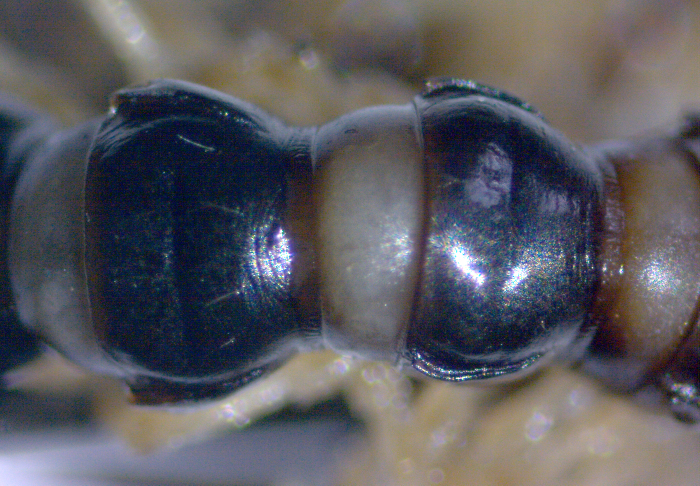
midbody segments, dorsal view

**Figure 1c.**
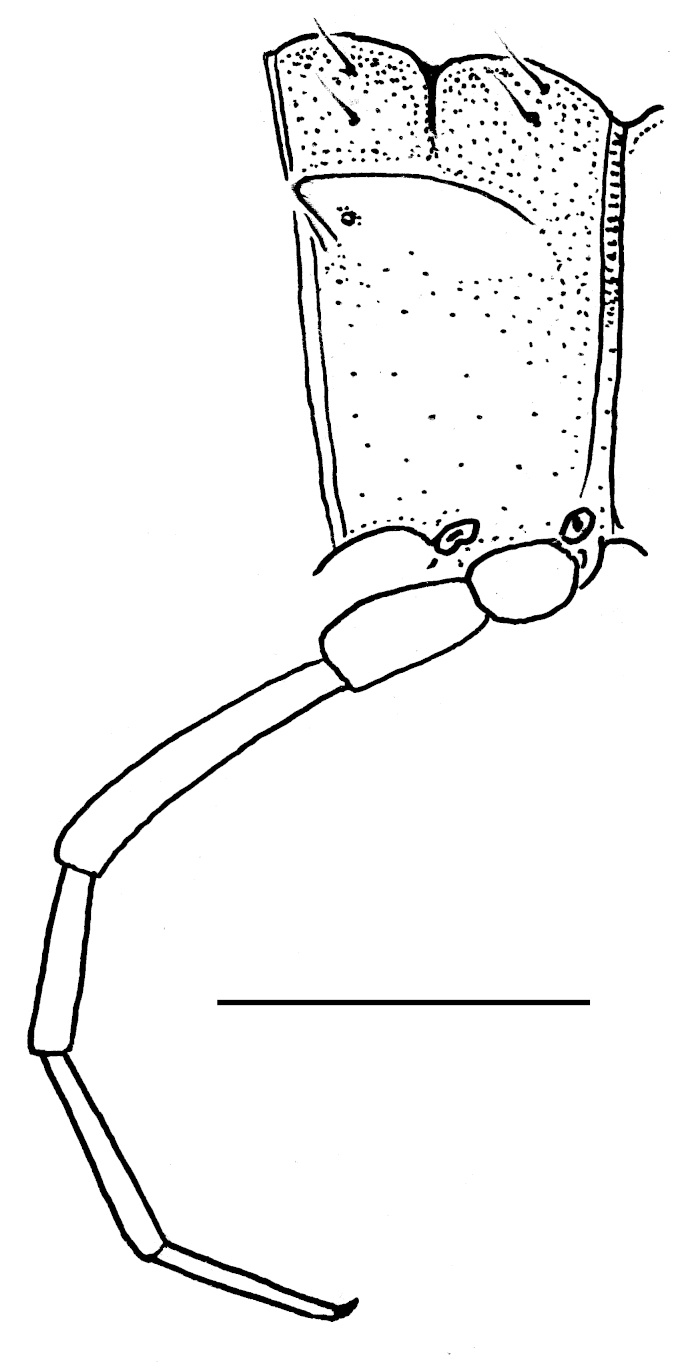
♂ paratype 3 segment 10, lateral view

**Figure 1d.**
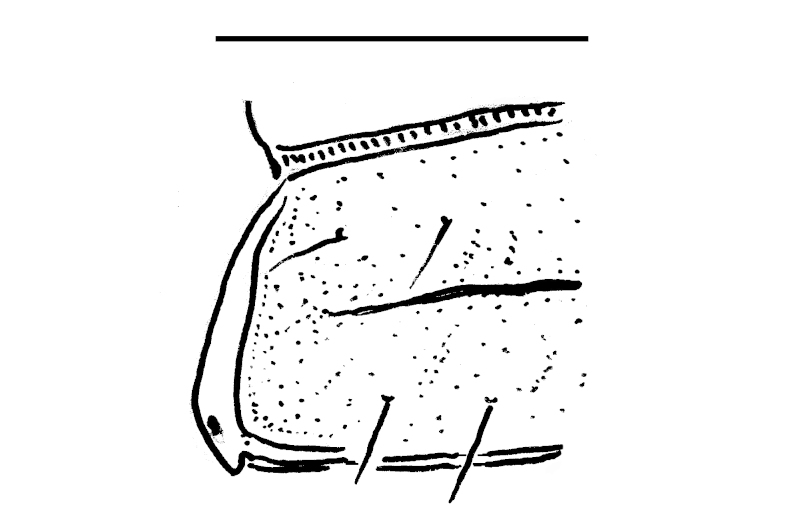
left half of segment 10, dorsal view

**Figure 2a.**
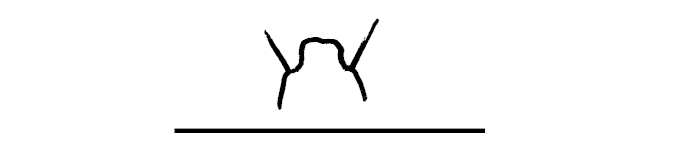
epiproct

**Figure 2b.**
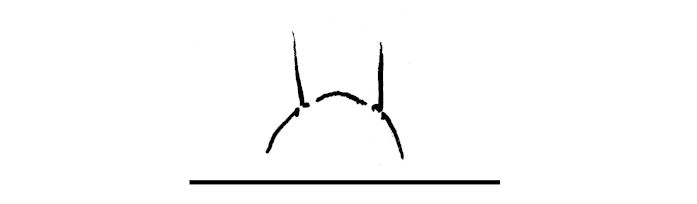
hypoproct

**Figure 2c.**
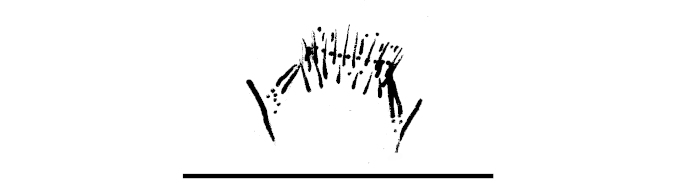
sternal lobe between coxae 4, caudal view

**Figure 2d.**
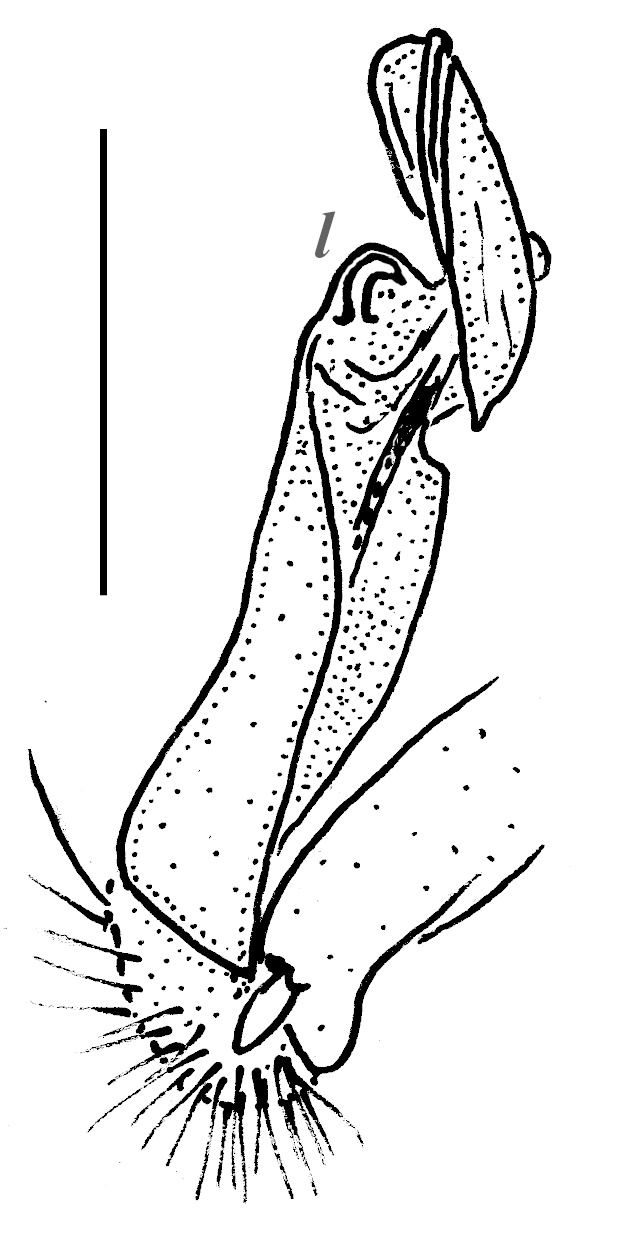
right gonopod, mesal view

**Figure 2e.**
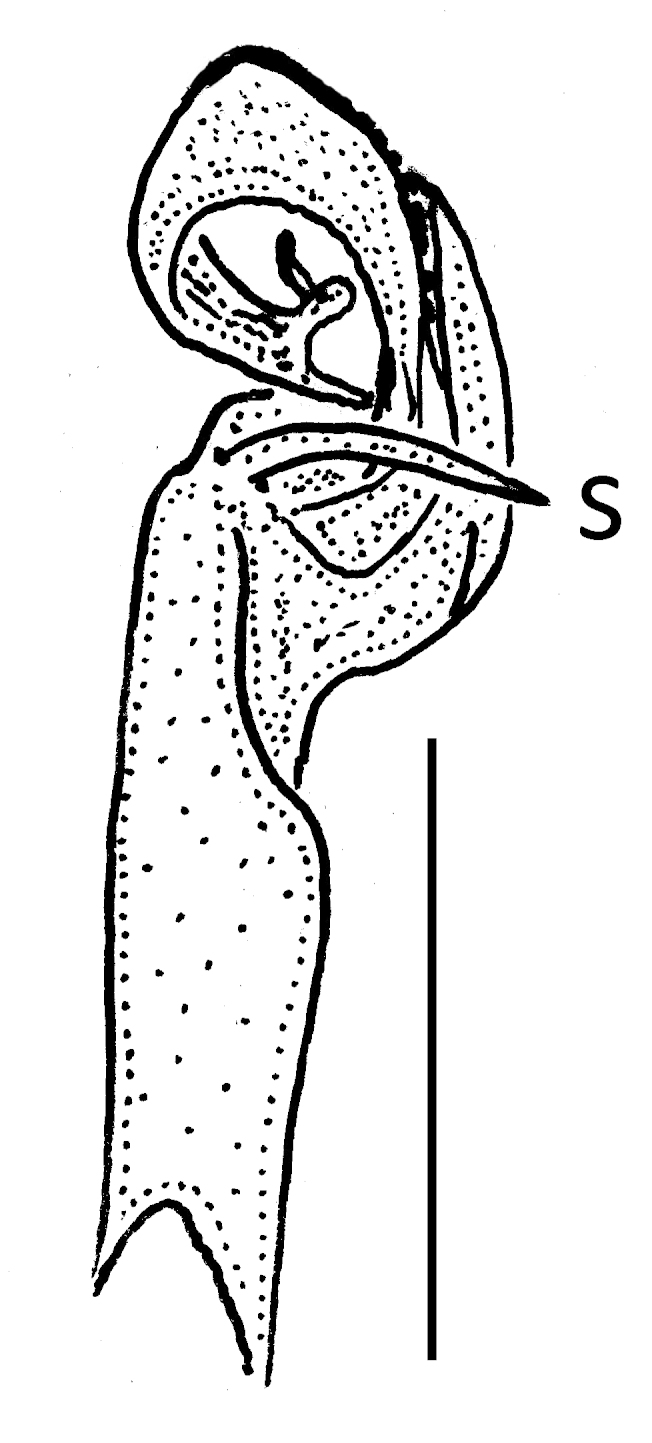
right gonopod, lateral view

**Figure 2f.**
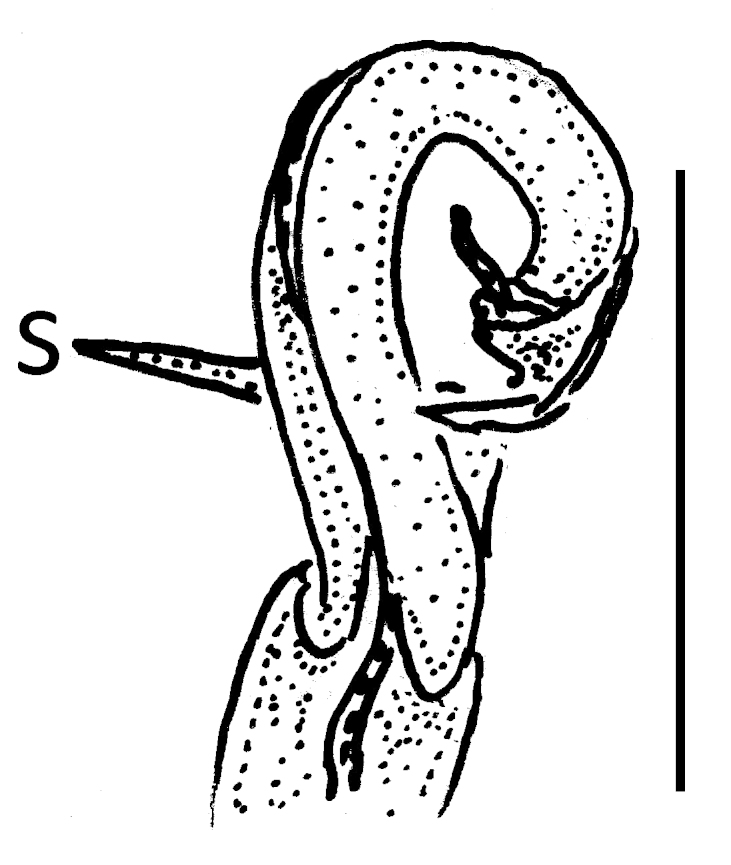
right gonopod, dorsal view

**Figure 5a.**
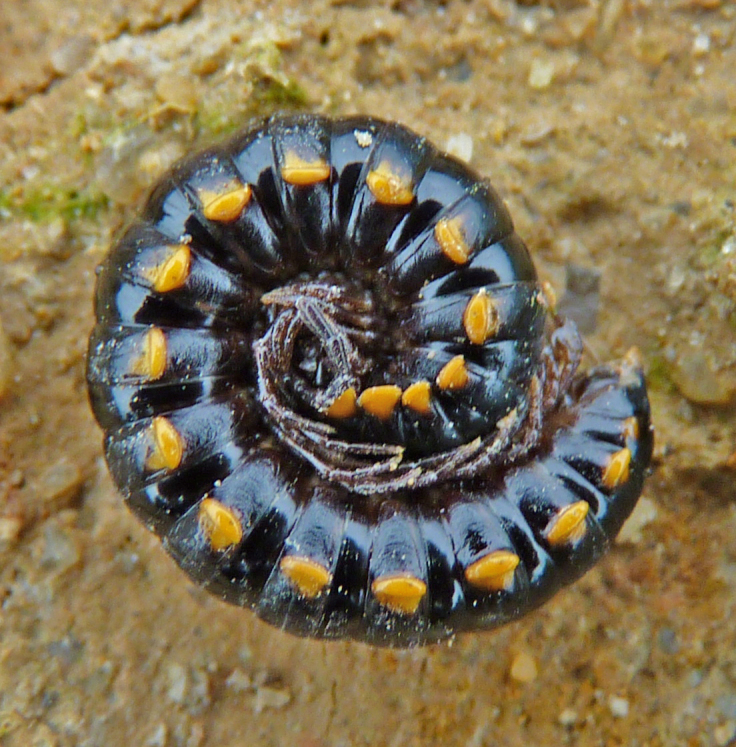
habitus, lateral view

**Figure 5b.**
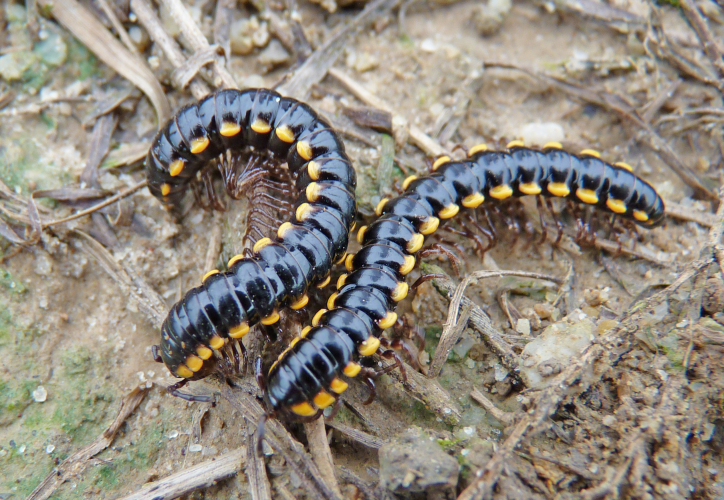
habitus, in situ, dorsal view

**Figure 6a.**
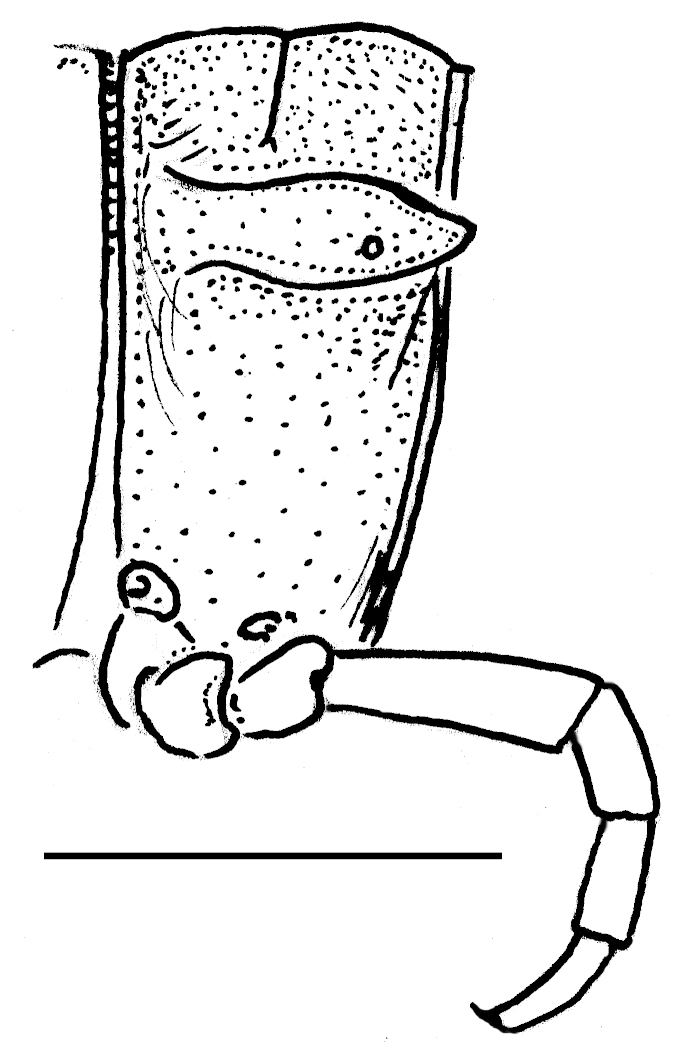
segment 10, lateral view. Scale bar: 2.0 mm

**Figure 6b.**
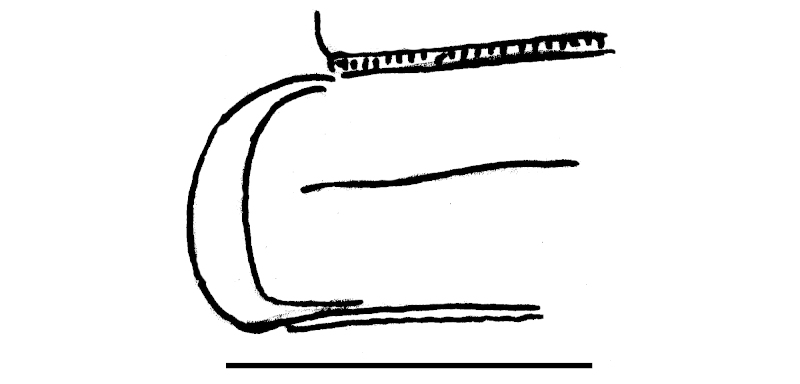
left half of segment 10, dorsal view. Scale bar: 2.0 mm

**Figure 6c.**
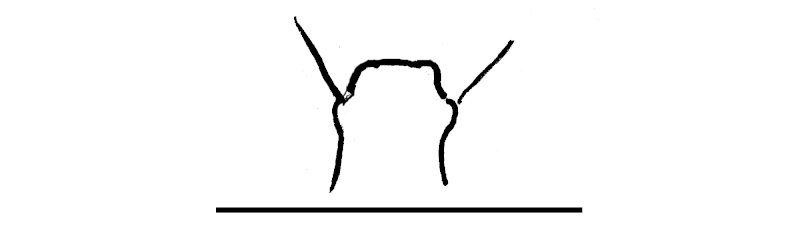
epiproct. Scale bar: 2.0 mm

**Figure 6d.**
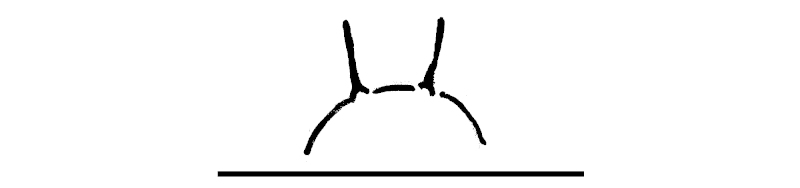
hypoproct. Scale bar: 2.0 mm

**Figure 6e.**
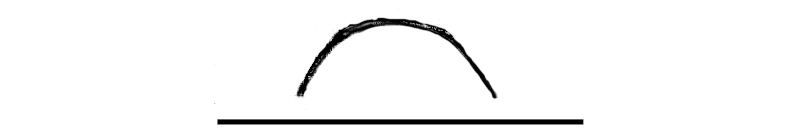
sternal lobe between coxae 4, caudal view. Scale bar: 2.0 mm

**Figure 6f.**
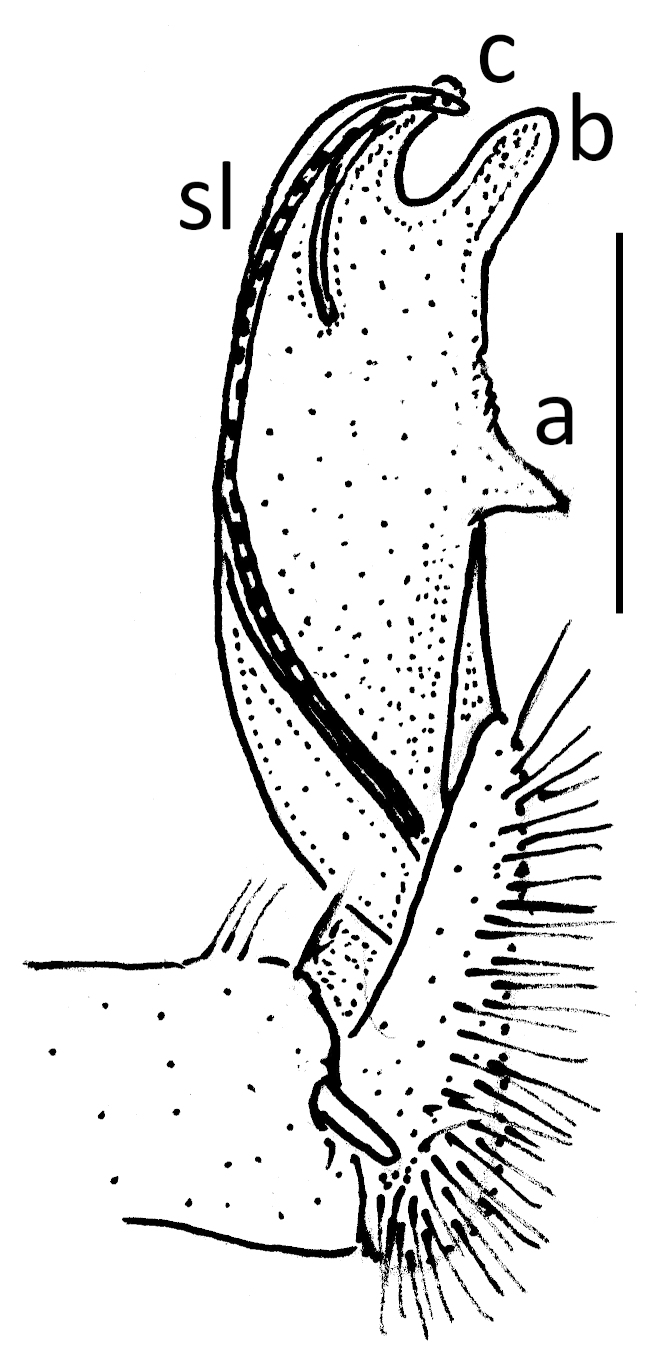
right gonopod, mesal view. Scale bar: 1.0 mm

**Figure 7a.**
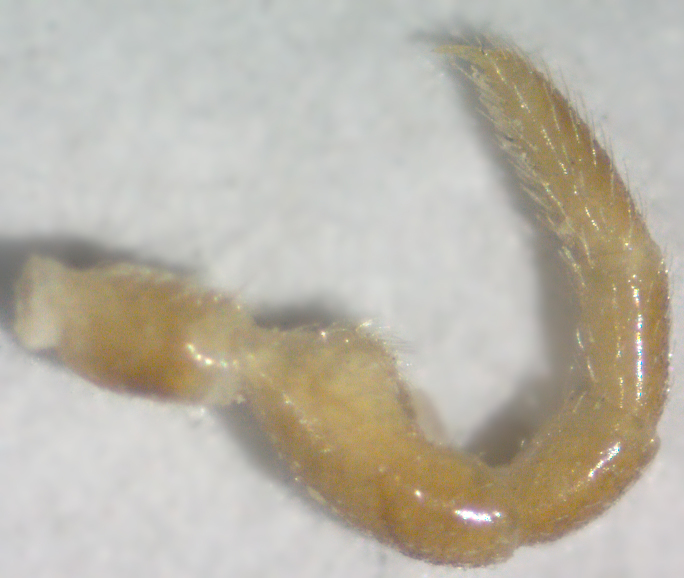
male leg 5, lateral view

**Figure 7b.**
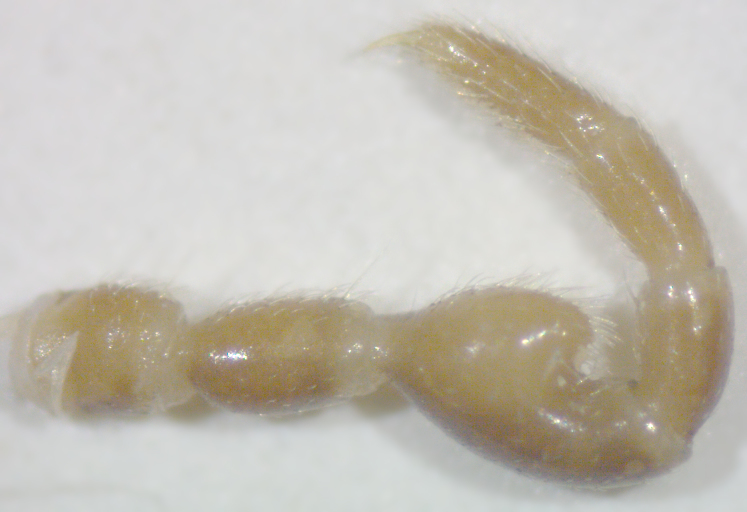
male leg 7, lateral view
